# Future Perspectives of NMDAR in CNS Disorders

**DOI:** 10.3390/molecules30040877

**Published:** 2025-02-14

**Authors:** Toni Capó, Joan Biel Rebassa, Iu Raïch, Jaume Lillo, Pau Badia, Gemma Navarro, Irene Reyes-Resina

**Affiliations:** 1 Department of Biochemistry and Physiology, Faculty of Pharmacy and Food Sciences, University of Barcelona, 08028 Barcelona, Spain; tonicapoquetglas@ub.edu (T.C.); jrebaspa7@alumnes.ub.edu (J.B.R.); iraichpa7@ub.edu (I.R.); pbadiafe47@alumnes.ub.edu (P.B.); 2 Centro de Investigación en Red, Enfermedades Neurodegenerativas (CIBERNED), Instituto de Salud Carlos III, 28031 Madrid, Spain; jaumelillo@ub.edu; 3 Institute of Neuroscience, University of Barcelona (NeuroUB), Campus Mundet, Passeig de la Vall d’Hebron171, 08035 Barcelona, Spain; 4 Department of Biochemistry and Molecular Biomedicine, School of Biology, University of Barcelona, 08028 Barcelona, Spain

**Keywords:** NMDA receptors, Alzheimer’s disease, Parkinson’s disease, ischemic stroke, schizophrenia

## Abstract

Neurodegenerative diseases such as Alzheimer’s and Parkinson’s diseases are among the leading causes of physical and cognitive disability across the globe. Fifty million people worldwide suffer these diseases, and that number is expected to rise as the population ages. Ictus is another pathology that also courses with neurodegeneration and is a leading cause of mortality and long-term disability in developed countries. Schizophrenia is not as common as other mental disorders, affecting approximately 24 million people worldwide. All these disorders have in common that still there is not an effective pharmacological treatment to cure them. The N-methyl-D-aspartate (NMDA) receptor (NMDAR) has attracted attention as a potential therapeutic target due to its important role in learning and memory and also due to its implication in excitotoxicity processes. Some drugs targeting NMDARs are already being used to treat symptoms of disorders affecting the central nervous system (CNS). Here, we aim to review the implications of NMDAR in these CNS pathologies, its role as a potential therapeutic target, and the future perspectives for developing new treatments focused on these receptors.

## 1. Introduction

Neurodegenerative disorders represent a major challenge for both medicine and public health in the coming years, given the aging of the population. These disorders are primarily characterized by the degeneration or loss of neurons. This neuronal degeneration results in a range of neuropsychiatric issues and permanent disability in affected individuals [1]. The most common neurodegenerative diseases include progressive neuropsychiatric conditions, such as Alzheimer’s disease (AD) and Parkinson’s disease (PD), among others [2]. Despite progress in biomedical science, molecular biology, genetics, and pharmaceutical sciences, significant challenges remain in the identification of effective therapies for these diseases, as many of them nowadays do not have a cure, and only symptomatic treatments are available for patients [1,2].

A common hallmark of many neurodegenerative diseases is the alteration in the expression and function of NMDA receptors [3,4,5,6]. NMDARs are glutamate-gated ion channels, with an important role in synaptic plasticity, learning, and memory, that have also been related to neurodegeneration and excitotoxicity [6,7,8]. NMDAR dysfunctions are also involved in various neurological and psychiatric disorders, including stroke, pathological pain, and schizophrenia; thus, there is growing interest in developing new drugs that target these receptors [9]. Here, we aim to review the implication of NMDAR in different disorders affecting the central nervous system (CNS), such as neurodegenerative diseases, ischemic stroke, or psychosis.

## 2. Structure, Expression, and Mobility of NMDARs

Glutamate is the main excitatory neurotransmitter in the CNS [10]. Glutamate acts on various membrane receptors, classified in metabotropic and ionotropic receptors. Ionotropic glutamate receptors are ligand-gated ion channels which are activated by glutamate, and they are mainly classified in α-amino-3-hydroxy-5-methyl-4-isoxazolepropionic acid (AMPA), kainite, and NMDA receptors [11].

**Figure 1:**
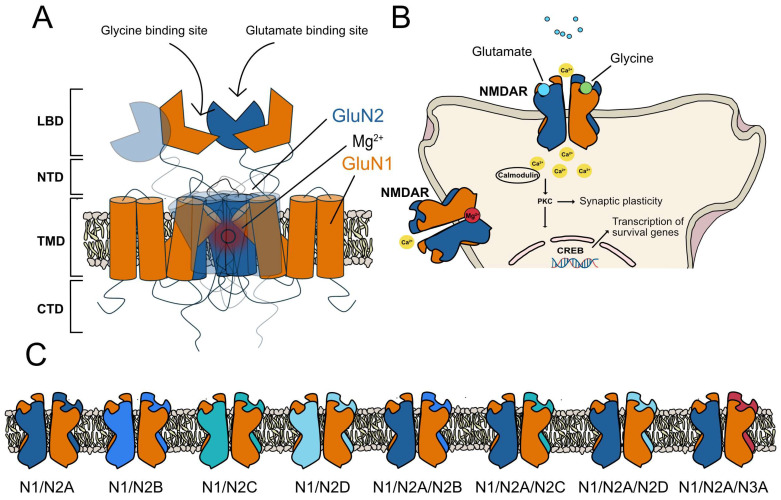
NMDAR structure, function, and subunit diversity. (**A**) Structure of the NMDA receptor, showing the ligand-binding domain (LBD) and N-terminal domain (NTD), where several allosteric modulators bind, altering the NMDAR functionality, transmembrane domain (TMD), and C-terminal domain (CTD). (**B**) The activation of NMDAR has multiple physiological effects, as this receptor plays a key role in neurotransmission, synaptic plasticity, and neuronal survival. (**C**) NMDARs are tetrameric receptors formed by the combination of different subunits: the GluN1 subunit, four different GluN2 subunits (GluN2A, GluN2B, GluN2C, and GluN2D), and two GluN3 subunits (GluN3A and GluN3B), all of them encoded by different genes.

NMDARs are tetrameric receptors formed by the combination of different subunits: the GluN1 subunit, four different GluN2 subunits (GluN2A, GluN2B, GluN2C, and GluN2D), and two GluN3 subunits (GluN3A and GluN3B), all of them encoded by different genes [9]. Usually, NMDAR tetramers are composed by two obligatory GluN1 subunits plus two GluN2 and/or GluN3 subunits (Figure 1). The presence of a wide variety of homologous NMDAR subunits enables different subunit combinations, leading to a diverse range of receptor subtypes in the central nervous system (CNS). These subtypes exhibit distinct biophysical and pharmacological characteristics, interact with different partners, and are localized in different subcellular compartments. The composition of subunits varies across different regions of the CNS, during development, and along disease states [10,12,13]. Depending on the combination of GluN2B subunit types, NMDARs are classified as di-heteromeric, such as GluN1/GluN2B and GluN1/GluN2A receptors, or as tri-heteromeric, e.g., GluN1/GluN2A/GluN2B receptors [9].

As seen in Figure 1, the NMDA receptor is a tetrameric receptor composed of main domains: the extracellular ligand-binding domain (LBD), where glutamate (on GluN2) and glycine/D-serine (on GluN1) bind to activate the receptor; the N-terminal domain (NTD), which modulates receptor function and allosteric regulation; the transmembrane domain (TMD), forming the ion channel pore; and the intracellular C-terminal domain (CTD), involved in signaling and receptor trafficking [9]. The TMD contains the Mg^2+^ blockade site, where, at resting membrane potential, extracellular Mg^2+^ ions block the ion channel, preventing ion flux. Upon sufficient depolarization, the Mg^2+^ block is relieved, allowing Na^+^ and Ca^2+^ influx and K^+^ efflux, crucial for synaptic plasticity and excitatory neurotransmission. The LBD and NTD are critical for modulating receptor activity, as they contain binding sites for agonists, antagonists, and allosteric modulators, making them important targets for pharmacological intervention in neurological disorders [9]. The crystal structure of GluN1A-GluN2B NMDA receptors is found in the Protein Data Bank under accession code 4PE5 [14].

The GluN2A and GluN2B subunits show different spatiotemporal expression profiles. In rodents, GluN2B is widely expressed in the embryonic brain, while GluN2A expression starts shortly after birth. Afterwards, GluN2A expression progressively increases and becomes abundant throughout the central nervous system, while GluN2B expression is maintained at high levels following birth, peaks around the first postnatal week, and becomes progressively restricted to the forebrain [15,16,17]. Thus, during early postnatal development, NMDARs switch their subunit composition from mainly containing GluN2B subunits to predominantly containing GluN2A subunits. This developmental switch is evolutionarily conserved and takes place in many areas of the brain, such as the cortex, hippocampus, amygdala, and cerebellum. This replacement of GluN2B with GluN2A subunits is not absolute, as in many regions of the adult CNS, GluN2B subunits are still expressed [9,15]. The subunit composition of NMDARs is not static; it changes during development in response to neuronal activity or sensory experiences, and this plasticity does not only occur during development but also in adult synapses [9].

Di-heteromeric GluN1/GluN2B and GluN1/GluN2A receptors represent an important fraction of juvenile and adult NMDARs, while tri-heteromeric GluN1/GluN2A/GluN2B receptors also populate many regions in the adult brain, particularly in the hippocampus and cortex [9]. NMDA receptors are usually located at the postsynaptic density (PSD) in dendritic spines, although the expression of NMDARs has also been detected at presynaptic and perisynaptic sites [6]. Within individual neurons, the expression of different NMDAR subtypes is segregated in an input-specific manner. For example, in pyramidal neurons of the adult hippocampal CA3 region, GluN2B subunits are expressed in dendrites that receive inputs from the entorhinal cortex, but not from the dentate gyrus [18]. Also, in the pyramidal neurons of layer 5, GluN2A and GluN2B are differentially enriched at synapses of the callosal and the intracortical pathways, respectively [19]. NMDAR subtypes also vary according to subcellular localization, as in the adult forebrain, synaptic NMDARs are mainly di-heteromeric GluN1/GluN2A and tri-heteromeric GluN1/GluN2A/GluN2B receptors, while peri- and -extrasynaptic sites are enriched in GluN2B-containing receptors [6,20]. Thus, GluN2A subunits seem to be more prevalent at synaptic locations, whereas GluN2B can be found in both synaptic and extrasynaptic regions. NMDARs are highly dynamic at the membrane and likely move between synaptic and extrasynaptic areas through lateral diffusion [21]. Receptors containing GluN2B subunits diffuse more quickly than those with GluN2A subunits, which helps promote the higher concentration of GluN2A at mature synaptic locations [22].

The GluN2A and GluN2B subunits exhibit distinct gating and kinetic properties, meaning that the subunit composition influences the biophysical, pharmacological, and signaling characteristics of NMDARs. When agonists are fully bound, GluN1/GluN2A receptors have a higher open probability compared to GluN1/GluN2B receptors [10]. In pyramidal cells, synaptic NMDARs, which typically contain GluN2A subunits, exhibit faster kinetics, while extrasynaptic NMDARs, predominantly containing GluN2B subunits, show slower kinetics [23]. Thus, the ratio of GluN2B to GluN2A at synaptic sites plays a crucial role in determining the effects of NMDAR activation, including the overall calcium influx and subsequent signaling pathway activation. This ratio is not fixed; it changes in response to neuronal activity and sensory experiences during postnatal development, and it continues to vary in adult synapses [9].

Regarding synaptic plasticity, synaptic GluN2A-containing NMDARs have been described to be the main contributors to long-term potentiation (LTP), while extrasynaptic GluN2B-containing receptors make a larger contribution to the total charge transfer when long-term depression (LTD) is triggered [24]. GluN1/GluN2A/GluN2B triheteromeric channels, possibly the most abundant NMDARs in the adult forebrain, display intermediate levels of agonist sensitivity, channel open probability, and deactivation kinetics [25,26,27].

## 3. NMDAR Function: Role of NMDAR in Memory and Synaptic Plasticity

In the central nervous system of vertebrates, the primary mechanism of excitatory transmission is mediated by the neurotransmitter glutamate. As mentioned above, NMDARs are ionotropic glutamate receptors, and they are ligand-gated ion channels which are activated by glutamate [11]. NMDARs have an important role in synaptic plasticity, learning, and memory, as they are implicated in excitatory synaptic transmission and in the regulation of mechanisms such as LTP and LTD [28]. These receptors are capable of converting specific patterns of neuronal activity into long-term changes in the structure and function of synapses, which are believed to support higher cognitive functions [28]. NMDARs also play a role in the experience-dependent refinement of synaptic connections during development [9,10].

The activation of NMDARs requires the binding of glycine (or D-serine) to GluN1 subunits together with the binding of glutamate to GluN2 subunits [29]. During basal synaptic transmission, Mg^2+^ blocks the NMDAR channel and prevents it from opening. Thus, NMDARs contribute little to the postsynaptic response during basal synaptic activity. However, upon the slight depolarization of the postsynaptic cell, the Mg^2+^ block is relieved of the channel, allowing the channel to open, and both Na^+^ and Ca^2+^ flow into the dendritic spine. This intracellular Ca^2+^ increase initiates a complex signaling cascade of subsequent events that drive synaptic plasticity, such as the incorporation of more AMPA receptors in the postsynaptic membrane [30]. This rise in AMPA receptor activity boosts the excitatory current, making it more likely that the postsynaptic neuron will fire when the synapse is activated again. Together, AMPA and NMDA receptors provide a direct mechanism for Hebbian learning, illustrating how the simultaneous firing of neurons can enhance synaptic strength [31]. Due to the need for simultaneous presynaptic glutamate release and postsynaptic depolarization, NMDARs are regarded as Hebbian coincidence detectors.

Thus, while AMPARs mediate fast excitatory transmission, NMDAR activation leads to long-term plasticity, which is expressed as changes in AMPAR-mediated transmission. These changes consist of either synaptic strengthening or weakening, taking place through the established processes of long-term potentiation (LTP) and long-term depression (LTD), thereby serving as the molecular substrate for learning and memory [9].

NMDAR-dependent LTP is one of the most studied forms of synaptic plasticity and it is known to contribute to the formation of long-term memory in the hippocampus. Besides being associated with learning and memory processes [32,33], it has also been related to clinical recovery after brain damage [34]. Upon NMDAR activation, calcium concentration likely has to reach a critical threshold value to activate the intracellular events necessary for LTP [35,36]. LTP consists of a lasting increase in synaptic excitability, accompanied by structural changes at both the presynaptic and postsynaptic terminals [37,38]. The induction of LTP is also linked to the remodeling of dendritic spines, which includes increases in spine volume, stability, and clustering [39].

The activation of NMDARs can also induce a different form of plasticity, namely long-term depression (LTD), which is associated with a lasting reduction in synaptic excitability [40]. LTD occurs when only a modest increase in postsynaptic calcium concentration within dendritic spines takes place, due to a modest activation of NMDARs [41]. This leads to the preferential activation of protein phosphatases, resulting in AMPARs abandoning the post-synapse, followed by a marked spine shrinkage, ultimately leading to the elimination of dendritic spines [42]. There is growing evidence that LTD mechanisms play a role in a variety of brain processes, including experience-dependent development, learning and memory, addiction, and neurological disorders like Alzheimer’s disease and Parkinson’s disease [40,43,44,45].

Although they have opposing effects, LTP and LTD work together to refine neural connections during development and regulate cognitive processes.

## 4. Role of NMDAR in Alzheimer’s Disease

Alzheimer’s disease is considered a neurodegenerative disease and, nowadays, is the main cause of dementia [46]. It is characterized by the deposition of abnormal protein aggregates, particularly in the hippocampal and cortical regions of the brain, which are involved in the memory and learning processes [47]. It has been demonstrated that the extracellular accumulation of the β-amyloid (Aβ) peptide and the presence of intracellular neurofibrillary tangles (NFTs) induce a neurodegeneration process correlated with the loss of synaptic plasticity typical of AD patients [48,49]. The NMDAR plays a pivotal role in synaptic plasticity, learning, and memory processes. However, it has been described that the excessive Ca^2+^ entry through NMDAR induces a progressive synaptic dysfunction and neurotoxicity that correlates to a decline in cognition and memory, as characteristic of AD [50]. Nowadays, there is no effective treatment to cure AD, however, the prescription of some drugs has been approved by the FDA. One of these drugs is memantine, a noncompetitive NMDAR antagonist used for treating moderate to severe AD patients [51].

This drug overlaps with the magnesium site, thereby inhibiting glutamate binding to the NMDAR [52]. Thus, memantine plays an important role in AD patients, who suffer from a dysregulation of the glutamatergic system due to the presence of the Aβ peptide. It has been described that the glutamate uptake and recycling system is severely weakened in AD patients. In this sense, a decrease in the protein expression of the glutamate transporter and a loss of the vesicular glutamate transporter (VGluT) have been shown [53]. Moreover, the excitatory amino acid transporter 2 (EAAT2), which is located in perisynaptic astrocytes and plays an important role in the glutamatergic cycle in the CNS, has an impaired function in the presence of the Aβ peptide [54]. Taking all into account, the presence of toxic Aβ levels allows more glutamate availability by impairing glutamate reuptake/recycling mechanisms. These increased glutamate levels hyperactivate the NMDAR, causing a stage of excitotoxicity and enhancing local neurodegeneration [55]. However, the complete activation of the NMDAR requires not only the binding of the glutamate but also the binding of one of the two coagonists, either D-serine or glycine [56]. It has been demonstrated that the presence of the Aβ peptide not only modulates the electrophysiological function of the NMDAR but also the levels of the coagonists [57]. Furthermore, it has been shown that AD increases the expression of serine racemase and then the levels of D-serine [58]. Taking into consideration the deep implication of the Aβ peptide on the AD physiopathology, several anti-Aβ antibodies have been recently approved, such as aducanumab in 2021, lecanemab in 2023, and donanemab in 2024 [59]. While lecanemab was distinctive for having tenfold stronger binding to protofibrils compared to fibrils, aducanumab showed a preferent binding to fibrils over protofibrils [60]. Donanemab is a humanized IgG1 antibody that specifically targets an N-terminal pyroglutamate Aβ epitope, which is exclusively found in mature amyloid plaques. It exhibits high selectivity for this epitope and does not bind to other Aβ species, thus presenting no off-target effects [61].

On the other hand, some studies have pointed out the important alterations of the release machinery of the presynaptic neurons in AD [62,63]. In later stages of the disease, it has been observed that the presence of the Aβ peptide induces a significant reduction in the expression of many components of the neurotransmitter release machinery, such as syntaxin, synaptophysin, and synaptotagmin proteins [57]. In this sense, the availability of glutamate is being compromised, which promotes the pathological synaptic loss of the disease. Moreover, it has been described that in AD, there is an inhibition of LTP in the hippocampus that alters the strength of synaptic transmission [64].

However, emerging evidence indicates that the membranous location of NMDAR, either synaptic or extrasynaptic, may be key to AD pathophysiology [65]. NMDARs are highly mobile, and a tilted equilibrium between synaptic and extrasynaptic NMDAR activity has been shown to contribute to neuronal dysfunction characteristic of some chronic neurodegenerative diseases, such as Alzheimer’s disease. Evidence reports that while synaptic GluN2A-containing NMDAR activation mediates LTP and promotes neuroprotective action, the Ca^2+^ entry through extrasynaptic GluN2B-containing NMDAR activation induces LTD and triggers cell death [6].

What occurs during AD at the synapse is well established. The presence of the Aβ peptide induces a synaptic depression resulting from an initial increase in synaptic activation by glutamate, followed by a desensitization and internalization of synaptic NMDARs, and finally, the activation of extrasynaptic or perisynaptic GluN2B-NMDARs, that have a key role on LTD [66]. While synaptic GluN2A-NMDARs induce the activity of CREB, which is involved in neuroprotective action, the extrasynaptic GluN2B-NMDAR triggers the CREB shut-off pathway related to pro-death and oxidative stress signaling and activates FOXO transcription factors [8,67].

Moreover, tau protein, the component of the NFT present in AD, has been involved in the regulation of synaptic function. It has been demonstrated that tau protein is required for Fyn-mediated GluN2B-containing NMDAR activation, which enhances its activity, inducing excitotoxicity [68]. In addition, some studies observed that the reduction in tau protein inhibited the excitotoxicity induced by glutamate, an event that was exacerbated by overexpressing tau [69,70]. Recently, it has been demonstrated that activating extrasynaptic NMDAR induces an overexpression of tau protein, which promotes neuronal degeneration and cell death [71].

In 2024, the Alzheimer’s disease drug development pipeline included 164 clinical trials evaluating a total of 127 promising drug candidates, highlighting the ongoing efforts to advance treatment options for this condition [72]. Among the Phase 3 candidates, 34% target neurotransmitter receptors, 22% target amyloid-related processes, and 12% are focused on synaptic plasticity/neuroprotection [72].

To summarize, the main drugs currently used for Alzheimer’s disease are cholinesterase inhibitors, which target acetylcholinesterase (AChE) and, in some cases, butyrylcholinesterase (BuChE), such as Donepezil, Rivastigmine, and galantamine; memantine, an NMDA receptor antagonist; and anti-Aβ peptide antibodies, such as aducanumab, lecanemab, and donanemab.

In conclusion, extrasynaptic NMDARs have an important role in the neurodegenerative stage of AD [73]. While memantine is administered in AD patients to block the activation of the extrasynaptic NMDAR, it also acts on the GluN2A-NMDARs, impairing their neuroprotective effects [74]. Therefore, current research focuses on finding new selective drugs to disrupt the extrasynaptic GluN2B-NMDAR activity without altering the GluN2A-NMDARs’ synaptic function.

## 5. Role of NMDAR in Parkinson’s Disease

Parkinson’s disease (PD) is the second most prevalent neurodegenerative disorder, affecting approximately 2–3% of individuals over the age of 65. PD is characterized by two main neuropathological features: significant neuronal loss in the substantia nigra, resulting in striatal dopamine deficiency, and the presence of intracellular inclusions containing α-synuclein aggregates, known as Lewy bodies [75]. These pathological alterations disrupt multiple cellular processes, including mitochondrial function, which leads to oxidative stress and neuroinflammation, but also α-synuclein proteostasis, calcium homeostasis, and axonal transport mechanisms [76]. These pathogenic mechanisms are responsible for the clinical symptoms of Parkinson’s disease, which include motor disturbances (especially resting tremor and bradykinesia), rapid eye movement (REM) sleep behavior disorder, and both autonomic and cognitive impairments [77].

Despite significant advances in understanding the pathophysiology of PD, the disease remains incurable and inevitably leads to severe disability. Current therapeutic approaches focus on symptomatic management, mainly through the use of the dopamine precursor levodopa (L-DOPA), dopamine receptor agonists, and monoamine oxidase inhibitors [78].

L-DOPA is still the most effective treatment available for ameliorating motor symptoms in PD patients, demonstrating sustained efficacy over multiple years of treatment in most cases. However, the prolonged use of L-DOPA is associated with the development of abnormal involuntary movements, termed L-DOPA-induced dyskinesia (LID) [79]. These motor complications are manifested as choreo-athetotic movements, dystonia, and ballism, potentially becoming as debilitating as the primary symptoms of PD [80].

Recent evidence suggests that the motor disturbances associated with L-DOPA arise from a dysfunction in the glutamatergic system of the basal ganglia [81]. The increased levels of glutamate observed in PD pathophysiology result in aberrant NMDA receptor stimulation, leading to increased intracellular calcium concentrations, mitochondrial dysfunction, oxidative stress, and ultimately cellular atrophy and death—a phenomenon known as glutamate-induced excitotoxicity [82].

The correlation between glutamatergic dysfunction and motor complications is further evidenced by specific alterations in NMDA receptor composition. Several studies have demonstrated that both dopaminergic neuronal loss and L-DOPA treatment induce a redistribution of NMDA receptor subunits [3,83]. Specifically, increased expression of GluN2A subunits and elevated GluN2A/GluN2B ratios have been observed in PD patients and in levodopa-treated dyskinetic animal models, including both rats and primates [84].

In addition to the changes in GluN2A/GluN2B ratios, elevated expression levels of the GluN1 and GluN2B subunits in the striatum of Parkinsonian rats were also observed [85]. Extensive research has revealed significant modifications in the NMDA receptor complex across both experimental models of Parkinsonism and human PD cases, suggesting a critical role for NMDA receptors in the molecular pathways underlying PD pathogenesis and subsequent clinical manifestations [86]. In fact, studies have shown that NMDA receptor hyperactivation in the striatum and nucleus accumbens contributes to the neurodegenerative acceleration in both experimental PD models and patient tissue samples [87,88]. This substantial body of experimental evidence supports the therapeutic potential of NMDA receptor antagonism in PD, with preclinical studies across diverse animal models demonstrating significant anti-Parkinsonian effects.

Multiple investigations have established that NMDA receptor antagonism effectively reduces catalepsy and attenuates rigidity and akinesia, among other motor symptoms in rodent PD models [89,90]. Furthermore, combining NMDA receptor antagonists with other compounds such as 7-nitroindazole and the opioid glycopeptide lactomorphin has demonstrated synergistic effects, enhancing the efficacy of L-DOPA while reducing the appearance of motor alterations associated with LID [91,92].

However, given the ubiquitous expression of NMDA receptors throughout the central nervous system and their fundamental role in physiological processes including synaptic plasticity, neural development, and learning, increasing evidence suggests that global NMDA receptor inhibition could lead to range of diverse adverse effects, including ataxia, cognitive impairment, and psychosis [93]. In this regard, current research efforts are focused on NMDA receptor subunit-selective antagonism. The NR2B subunit, which exhibits enhanced expression in the striatum and other basal ganglia regions in PD, has emerged as a promising therapeutic target for selective intervention in PD pathophysiology [85].

Selective NR2B antagonists, including ifenprodil and traxoprodil, have shown efficacy in reducing L-DOPA-induced dyskinesia in both rodent models and MPTP-lesioned primates [94,95]. Furthermore, NR2B antagonists have shown promising results in combination therapy approaches. For instance, the co-administration of radiprodil with the A_2A_R antagonist tozadenant resulted in enhanced motor performance in 6-OHDA-lesioned rats [96].

The metabotropic glutamate receptor 5 (mGluR5) exhibits increased expression in dopaminergic brain regions of Parkinson’s disease patients [97]. In addition, experimental evidence demonstrates that mGluR5 silencing effectively reduces L-DOPA-induced dyskinesia in murine PD models [98]. Recent investigations have elucidated that mGluR5 may contribute to PD pathophysiology through the enhancement of the activity of the NR2B-containing NMDA receptor through NR2B phosphorylation [99]. These findings suggest that a therapeutic approach combining NR2B antagonists with negative allosteric modulators of mGluR5 receptors could provide an effective strategy for mitigating NMDA-mediated excitotoxicity.

Despite promising preclinical results, clinical trials investigating glutamatergic modulation in PD patients remain in developmental stages. Currently, amantadine represents the only NMDA antagonist approved for clinical use in PD patients [100]. While a small-scale clinical study involving 13 PD patients demonstrated encouraging results for dextromethorphan in the treatment of L-DOPA-induced dyskinesia, this compound has not yet received FDA approval for this indication [101].

To summarize, the main drugs currently used for Parkinson’s disease are L-DOPA; monoamine oxidase inhibitors such as selegiline and asagiline, both targeting monoamine oxidase type B; dopamine agonists, like pramipexole and ropinirole, both targeting D_2_ and D_3_ receptors; adenosine agonists, targeting the A_2A_ receptor, such as istradefylline; and NMDA antagonists, such as amantadine.

Additional research on NMDA receptor antagonists and other glutamate receptor modulators, coupled with larger-scale clinical trials, is essential to validate these and find new, potential therapeutic targets for Parkinson’s disease treatment.

## 6. NMDA Receptors Are Related to Psychosis and Schizophrenia

Psychosis and schizophrenia are two related neurological disorders presenting a broad range of symptoms. In both, individuals’ perception of reality is altered. Thus, they may experience delusions, hallucinations, incoherent speech, and inappropriate behavior. This leads to difficulty in distinguishing what is real from what is not. It is estimated that between 15 and 100 of 100,000 people are being newly diagnosed with psychosis each year, highlighting its impact on society [102].

This disease originates around the mid-20s, when the brain and neuronal connections continue developing [102]. However, elderly people suffering from other neurological disorders are also at risk. Interestingly, it is possible to model/reproduce psychosis symptoms by treatment with different antagonists of NMDA receptors, such as phencyclidine (PCP) or ketamine [103,104,105]. Ketamine produces an increase in calcium ions after the NMDAR blockade, and the subsequent Ca^2+^ influx through non-NMDAR channels [106]. It is further suggested that ketamine also interacts with Plasma Membrane Calcium ATPase (PMCA) [107], and that NMDAR, PMCA, and PSD95 interact with each other, regulating calcium extrusion and NMDAR/PSD95 interaction [108]. The role of PCP is not fully understood, and the main hypothesis is that it blocks NMDAR, causing abnormal glutamate release [109].

Psychosis and schizophrenia are two pathologies that have been an object of study along the years on account of their prevalence, showing several characteristic causes and symptoms [102]. In the early 1970s, the “dopamine hypothesis of schizophrenia” was based on the fact that antipsychotics’ efficiency was directly correlated with the affinity to the dopamine D_2_ receptor. Later, elevations of dopamine in different regions of post-mortem brains from individuals with schizophrenia were reported, supporting the initial hypotheses. Nevertheless, reductions in the activity of glutamic acid decarboxylase and the reduced activity of choline acetyltransferase were determined, hence leading to the conclusion that the mechanism was not exclusively the dopaminergic signaling but rather a more complex fact [110]. Interestingly, a significant number of patients treated with antagonists of D_2_R never responded to the treatment, supporting this hypothesis [111]. In conclusion, the broad spectrum of psychosis cannot be attributed to just one receptor; on the contrary, it is linked to a more complex mechanism that involves more than one neurotransmitter, which could be simultaneously altered in the same patient.

A study by Mohn et al. (1999) tried to shed light on the NMDA receptor role in psychosis. They successfully knocked down the NR1 subunit of NMDA receptors in all cells to the 5% of its normotypical expression. These KD rats displayed behaviors similar to several schizophrenia animal models, revealing that a hypofunction of the NMDA receptor results in schizophrenic-like behavior [112].

There is clinical evidence that supports the hypofunction of NMDA receptors in psychosis or schizophrenia. Anti-NMDAR encephalitis is a condition characterized by the presence of antibodies against NMDA receptors, targeting the NR1subunit. These anti-NMDAR antibodies favor the internalization of NMDA receptors when bound to it. Consequently, patients exhibit psychotic-like symptoms [113], showing that a blockade of NMDA receptors contributes to psychotic/schizophrenic-like behavior. However, a study by Mallien et al. (2023) showed that in mainly GABAergic (Erb4 presenting neurons) neurons, the ablation of the NR1 subunit in adult stages is not capable of producing psychosis, nor other manifestations of neurological disorders. Thus, authors show that the absence of NR1 subunit in Erb4-presenting neurons cannot explain the symptoms of anti-NMDAR encephalitis in adult stages [114].

Regarding the other subunits present in the NMDA receptor complex, a study by Tarrés-Gatius et al. (2022) analyzed the involvement of the GluN2C subunit knockout. Interestingly, they found that GluN2C is mainly implicated in the motor incoordination provoked by the administration of pro-psychotic drugs (PCP and MK-801), as GluN2C KO mice did not present it upon the administration of PCP or MK-801. On the other hand, they did not find a relationship between this subunit and the sensorimotor gating (a relevant aspect of schizophrenia), as WT and KO mice exhibited identical responses to PCP and MK-801 on tests assessing this aspect [115]. On the other hand, using the GlucN2D knockout, a study by Vinnakota et al. provides evidence that the GlucN2D subunit is also involved in the hyperlocomotion of mice after the administration of R-norket, ketamine, or PCP [116].

Furthermore, Brakatselos et al. described that ketamine administration decreased NMDA receptor NR1 and NR2B subunit expression, supporting the hypothesis of NMDA hypofunction in psychosis [117]. Moreover, an important reduction in ERK phosphorylation was also observed. In the same study, mice were pretreated with cannabidiol (CBD). CBD was able to reverse NMDA subunit expression and ERK phosphorylation decline, showing higher NMDA expression in some regions, such as the nucleus accumbens, compared to controls. However, and very interestingly, the pre-administration of CBD did not reverse ketamine-induced hyperactivity but maintained it for a longer period, of which authors argue possible pharmacokinetic interactions or interplay with other receptors. These results show promise for relating cannabinoid properties to psychosis, even though the role of CBD is not fully understood due to controversy between different studies, as it may be dependent on the animal model and procedures developed [117,118]. However, it is clear that cannabinoids play an important role in NMDAR in psychosis and schizophrenia [119,120,121].

The main drugs currently used for psychosis and schizophrenia are the dopamine D_2_ receptor antagonist, such as haloperidol, risperidone, or clozapine; serotonin 5-HT_2A_ receptor antagonist, such as olanzapine or risperidone; or NMDA antagonists, like phencyclidine or ketamine.

In conclusion, it has been shown that NMDA is implicated in psychosis and schizophrenia. In spite of the variability of the results obtained due to the different models and protocols employed, the aforementioned studies indicate a broad implication of NMDAR in those pathologies and that the receptors may modify several mechanisms.

## 7. Role of NMDAR in Ictus

Stroke remains a leading cause of mortality and long-term disability in developed countries and represents a substantial global economic burden [122]. It is responsible for nearly 4% of total healthcare expenditures annually, placing a significant strain on healthcare systems [123]. Stroke is broadly classified into two main types: ischemic and hemorrhagic. Ischemic stroke accounts for approximately 87% of all cases [123] and is primarily caused by thrombotic or embolic occlusion of cerebral arteries. This arterial obstruction leads to a reduction in cerebral blood flow, resulting in energy depletion and initiating a cascade of complex pathophysiological processes. These include the disruption of ionic homeostasis, excessive synaptic and extrasynaptic glutamate accumulation, ion channel dysfunction, oxidative stress, membrane and DNA damage, as well as neuroinflammation, all of which culminate in neuronal cell death and ischemic brain injury [124].

Currently, the only FDA-approved pharmacotherapy for acute stroke is intravenous thrombolytic therapy with the recombinant tissue plasminogen activator (rtPA) [125]. However, rtPA is constrained by a narrow therapeutic window of 3–4.5 h and a 6–7% risk of intracerebral hemorrhage, limiting its use to approximately 5% of stroke patients [126]. This underscores the critical need for safer and more effective stroke therapies. Over recent decades, substantial research has deepened our understanding of stroke pathology, with NMDAR-mediated excitotoxicity remaining a central focus.

Excessive glutamate release, as noted earlier, is linked to neuronal excitotoxicity. This involves the overstimulation of certain ion channels in the postsynaptic neuron, such as NMDA and AMPA receptors. These channels mediate cationic inward currents in response to the glutamate released by presynaptic neurons or reversed uptake mechanisms in astrocytes [127]. The activation of NMDA receptors facilitates the influx of Na^+^ and Ca^2+^ ions. Elevated intracellular Ca^2+^ levels activate calpains, caspases, nitric oxide synthase, and enzymes that produce free radicals and arachidonic acid metabolites. These processes lead to protein cleavage, plasma membrane rupture, and the activation of the pro-apoptotic protein BID. Concurrently, the increase in Ca^2+^ and reactive oxygen species (ROS) triggers the opening of the mitochondrial permeability transition pore (MPTP), a large conductance channel within the inner mitochondrial membrane. The opening of the MPTP results in ATP depletion, loss of mitochondrial function, mitochondrial swelling, and the release of cytochrome C. These events culminate in the activation of apoptotic and necrotic pathways, ultimately causing cell death [128].

Neuronal death after stroke is a prolonged process [129], and understanding its mechanisms could enable therapies to reduce damage even when applied days later. While NMDAR-mediated excitotoxicity is a key contributor [130], selective NMDAR blockers are clinically impractical due to side effects and limited therapeutic windows [131]. Understanding the mechanisms by which NMDARs mediate the balance between neuronal survival and death remains a critical area of research, with recent studies proposing hypotheses to elucidate this dual role.

Given the central role of NMDARs in excitotoxicity, early therapeutic strategies are focused on receptor blockade [132]. NMDAR antagonists were developed to target specific sites: non-competitive antagonists that inhibit the ion channel, competitive antagonists that prevent excitatory neurotransmitters from binding to the glutamate recognition site, and glutamate release inhibitors that block presynaptic voltage-gated sodium (Na^+^) channels [133]. In preclinical studies using a rat model of middle cerebral artery occlusion (MCAO), these antagonists demonstrated neuroprotective effects against ischemic neuronal death [134,135]. The MCA can be occluded transiently or permanently in these models, simulating strokes of varying severity [136].

Despite their initial promise in rodent models, NMDAR antagonists have not successfully translated to clinical use for acute stroke treatment [124]. This translational failure is likely attributable to multiple factors [137], with two key limitations being their narrow therapeutic time window and dose-limiting safety concerns. NMDAR antagonists require administration either prior to or immediately after stroke onset to demonstrate efficacy [138]. Furthermore, these agents are associated with severe side effects, including nausea, vomiting, cardiovascular complications, and psychomimetic effects in treated patients [90].

Consequently, the lack of clinical success with NMDAR antagonists has prompted a shift in stroke neuroprotection research towards identifying and targeting downstream intracellular signaling pathways activated by NMDARs. The final goal of these emerging therapeutics is to selectively enhance neuroprotective signaling complex activity [139,140] and/or inhibit neurodegenerative signaling complex pathways [141,142] without affecting other NMDAR-associated signaling processes. Some examples of drugs that target a few cellular signaling pathways that are involved in the pathophysiology of stroke are the following: GluN2BCT1292-1304 (targeting DAPK1/GluN2BPSD95-nNOS), Geniposide (targeting GluN2A/AKT/ERK), or Stevioside (targeting TLR/NF-kB) [143]. By targeting downstream signaling events, these therapies provide a broader therapeutic window and improved clinical outcomes.

## 8. Current Treatments and Future Perspectives of NMDAR in CNS Disorders

**Figure 2:**
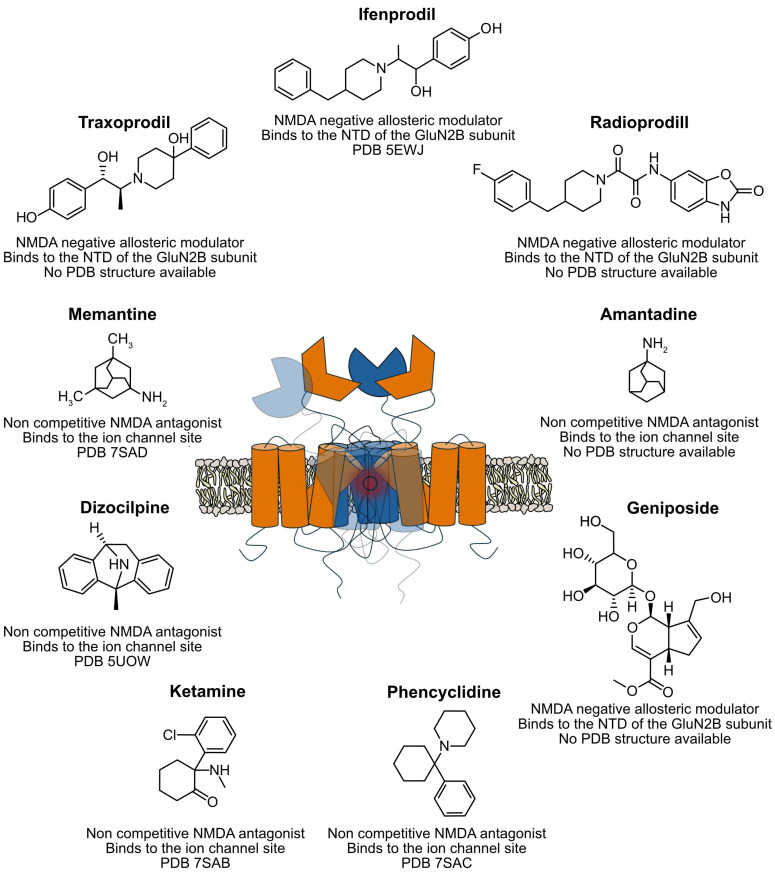
Schematic representation of the pharmacophores used for the treatment of different disorders targeting NMDRs. For each pharmacophore, the type of modulation it exerts on the receptor, its binding site on the NMDA receptor, and the corresponding PDB entry code (if available) describing its interaction with NMDA are provided.

Excitotoxicity has been identified as a key factor in the development of various neurodegenerative disorders. For this reason, NMDAR antagonists have been proposed as potential therapeutic agents to treat these conditions. These drugs are classified into three categories based on their mechanism of action: competitive, non-competitive, and negative allosteric antagonists [90] (Figure 2).

Competitive antagonists block the activation of the NMDA receptor by binding to the glutamate-binding site. Compounds such as D-CPP, D-CPP-ene (Midafotel), D-AP5, and D-AP7 have demonstrated anticonvulsant, anti-ischaemic, and antidepressant properties [144,145]. However, severe associated side effects, including hallucinations, confusion, paranoia, and even coma, have prevented these drugs from advancing in clinical trials [146].

Non-competitive antagonists, on the other hand, block ion conduction through the NMDA receptor channel independently of glutamate presence, which is why they are also known as channel blockers. This category includes compounds such as phencyclidine (PCP), dizocilpine maleate (MK-801), memantine, ketamine, and tiletamine. Despite their efficacy, these drugs have high receptor affinity, leading to rapid binding and slow dissociation. This prolongs calcium-mediated responses and may cause adverse effects, such as psychotomimetic symptoms and alterations in dopaminergic transmission, including schizophrenia-like symptoms in healthy individuals. These side effects have limited the clinical use of these antagonists. Despite these limitations, memantine and amantadine have marked a significant breakthrough in the treatment of neurodegenerative diseases due to their better clinical tolerability. Memantine, primarily used in the treatment of Alzheimer’s disease, selectively blocks excessive NMDA receptor activity without disrupting its normal physiological function, thereby improving patients’ cognition and daily activities [147]. Additionally, it has been shown to be effective in treating epilepsy. Amantadine, on the other hand, modulates dopamine release and uptake, alleviating motor symptoms such as rigidity, tremor, and levodopa-induced dyskinesia in Parkinson’s patients [148].

Other non-competitive antagonists, such as dextromethorphan and ketamine, have also demonstrated therapeutic benefits. Dextromethorphan, widely known as an active ingredient in cough medications, is also used to treat neurological symptoms like pseudobulbar affect, especially when combined with quinidine, marketed as Nuedexta [149]. Ketamine, initially developed as a dissociative anaesthetic, has been approved for treating treatment-resistant depression [150] and, in some cases, for relieving neuropathic pain [151]. However, ketamine and similar antagonists can interact with other receptors, such as those for dopamine, serotonin, AMPA, opioids, and GABA, potentially leading to adverse effects such as addiction, dependence, and tolerance, particularly with prolonged use [152].

Something that further complicates the pharmacology of these receptors is their well-described capacity to form complexes with other G protein-coupled receptors (GPCRs). Traditionally, it was assumed that GPCRs functioned as independent monomeric units, with a 1:1 stoichiometry with the trimeric G-protein. However, by the mid-1990s, accumulating evidence indicated that GPCRs could oligomerize. Heteromerization between GPCRs is defined as the direct interaction between at least two receptors, resulting in distinct biochemical and functional properties that differ from those of their individual components [153,154]. Therefore, the study of GPCR heteromers, particularly in the context of disease treatment, offers new opportunities for developing more targeted therapies, as these complexes may exhibit unique functional properties, even under pathological conditions. The development of drugs that can specifically target GPCR heteromers is crucial for advancing therapeutic strategies, especially in diseases where receptor interactions play a critical role in disease progression. Since heteromers exhibit unique biochemical and functional properties distinct from their individual receptor components, they may represent novel therapeutic targets that could offer greater specificity and efficacy. Targeting these complexes could allow for more precise modulation of signaling pathways, potentially reducing the side effects associated with targeting single receptors such as NMDA. Furthermore, in pathological conditions, where receptor functions can be altered, drugs that specifically modulate heteromeric complexes might offer a more effective way to restore or fine-tune receptor activity, thus providing better therapeutic outcomes in diseases such as Alzheimer’s, Parkinson’s, and other neurological or psychiatric disorders.

Some examples include the NMDAR-mGluR5 heterocomplexes, where mutual inhibition occurs between the two receptors, the NMDAR-D_1_R and NMDAR-D_2_R heterocomplexes, which play an important role in the GABAergic neurons of the striatopallidal pathway, or the heterocomplexes between NMDAR and cannabinoid receptors [155]. Specifically, the presence of the NMDAR-CB_2_R heterocomplex is increased in both microglia and neurons in APPSw/Ind transgenic mice, compared to the levels observed in control mice samples [121], which suggests that this heteromer could have a role in AD. In this regard, targeting the NMDAR-CB_2_R heterocomplex with a selective drug could offer several advantages for AD treatment. For instance, a pharmacological approach that selectively modulates the NMDAR-CB_2_R complex could potentially reduce the excitotoxicity caused by excessive glutamate signaling, while also mitigating neuroinflammation by influencing the CB_2_R’s role in immune cells, like microglia. Such a strategy could not only help preserve neuronal function but also alleviate some of the neurodegenerative processes that drive AD progression. On the other hand, it has been shown that the activation of CB_1_R in CB_1_R-NMDAR complexes may counteract the excessive overactivation of NMDAR in an Alzheimer’s mouse model, providing a dual beneficial effect by both offering cannabinoid signaling and reducing NMDAR activation [156]. Furthermore, as commented above, excessive NMDAR activation can lead to neuronal excitotoxicity and cell death, while excessive inhibition can negatively impact cognitive and neuronal function; thus, it is essential to develop therapeutic strategies that allow for more precise control of its activity. In this regard, allosteric modulators of NMDAR represent a promising option, as they regulate receptor function more selectively compared to orthosteric antagonists, which can cause severe side effects by completely blocking receptor-mediated signaling. Looking ahead, research on NMDAR allosteric modulators will focus on developing more specific compounds with optimized safety profiles, targeting particular receptor subpopulations, such as those forming heteromers with other receptors, including GPCRs. Identifying specific allosteric sites in different NMDAR subunits, such as GluN2A or GluN2B, will enable the design of drugs that selectively modulate receptor activity in specific brain regions, minimizing adverse effects.

In conclusion, targeting NMDA-containing heteromers, and the use of allosteric modulators, may present novel therapeutic opportunities for modulating NMDAR activity, and the development of bitopic drugs, capable of acting simultaneously on multiple receptor sites or different receptor types interacting with NMDAR, could open new opportunities in the treatment of neurological and psychiatric diseases.

## Data Availability

Not applicable.
